# Association of Troponin T levels and functional outcome 3 months after subarachnoid hemorrhage

**DOI:** 10.1038/s41598-021-95717-w

**Published:** 2021-08-09

**Authors:** Aida Anetsberger, Bettina Jungwirth, Manfred Blobner, Florian Ringel, Isabell Bernlochner, Markus Heim, Ralph Bogdanski, Maria Wostrack, Gerhard Schneider, Bernhard Meyer, Martin Graeßner, Lea Baumgart, Jens Gempt

**Affiliations:** 1 Department of Anesthesiology, Klinikum Rechts der Isar, Technische Universität München, Munich, Germany; 2 Department of Anesthesiology, Universitätsklinikum Ulm, Ulm, Germany; 3 Department of Neurosurgery, Universitätsmedizin Mainz, Langenbeckstr.1, 55131 Mainz, Germany; 4 I. Medizinische Klinik und Poliklinik, Klinikum Rechts der Isar, Technische Universität München, Munich, Germany; 5 Department of Neurosurgery, Klinikum Rechts der Isar, Technical University Munich, Ismaninger Str.22, 81675 Munich, Germany

**Keywords:** Neuroscience, Neurology

## Abstract

TroponinT levels are frequently elevated after subarachnoid hemorrhage (SAH). However, their clinical impact on long term outcomes still remains unclear. This study evaluates the association of TroponinT and functional outcomes 3 months after SAH. Data were obtained in the frame of a randomized controlled trial exploring the association of Goal-directed hemodynamic therapy and outcomes after SAH (NCT01832389). TroponinT was measured daily for the first 14 days after admission or until discharge from the ICU. Outcome was assessed using Glasgow Outcome Scale (GOS) 3 months after discharge. Logistic regression was used to explore the association between initial TroponinT values stratified by tertiles and admission as well as outcome parameters. TroponinT measurements were analyzed in 105 patients. TroponinT values at admission were associated with outcome assessed by GOS in a univariate analysis. TroponinT was not predictive of vasospasm or delayed cerebral ischemia, but an association with pulmonary and cardiac complications was observed. After adjustment for age, history of arterial hypertension and World Federation of Neurosurgical Societies (WFNS) grade, TroponinT levels at admission were not independently associated with worse outcome (GOS 1–3) or death at 3 months. In summary, TroponinT levels at admission are associated with 3 months-GOS but have limited ability to independently predict outcome after SAH.

## Introduction

Cardiac irregularities after subarachnoid hemorrhage (SAH) are common and include a wide range of manifestations like hyper- and hypotension, arrhythmias, myocardial injury or cardiac failure^1,2^. A majority of SAH patients develops electrocardiogram (ECG)—changes and more than 50% have increased Troponin levels^3^. Besides, some patients develop a severe form of stress-induced cardiomyopathy, neurogenic stunned myocardium (NSM) characterized by depressed ventricular function and regional wall motion abnormality (RWMA)^4^.

Numerous studies have explored the association of these findings with complications and outcome^3,5,6^. RWMAs, elevated NT-proBNP levels as well as some ECG changes, for example ST-segment depression, were significantly associated with an increased risk of death^7^. Although the elevation of cardiac troponins has been the topic of several investigations, its impact on outcome remains unclear. While there are studies linking cardiac troponin elevation with long-term outcome^8^, the association was not present after discharge in other trials^3^. Therefore, the aim of this study was to evaluate whether hsTroponinT values after SAH are associated with functional outcome at 3 months.

## Methods

### Study design

Troponin T measurements were obtained prospectively in the frame of a randomized, controlled clinical trial exploring the association of goal-directed hemodynamic therapy and outcomes after SAH at a University Hospital in Germany (Klinikum rechts der Isar, Technical University Munich)^9^. The study was approved by the local ethics committee (Ethics Commission of the Medical faculty, Technical University of Munich, ID: 5425-12) and was first registered at 16/04/2013 at clinicaltrials.gov; Identifier: NCT01832389. All research was performed in accordance with the Declaration of Helsinki.

Patients older than 18 with an aneurysmal SAH diagnosed by CT and confirmed by angiography were eligible for enrollment. Exclusion criteria were: traumatic SAH, congestive heart failure, severe diseases of aorta or aortic valve, pregnancy, calcium antagonist intolerance. Written consent was obtained from each patient or their legal representative. Clinical management was performed according to current guidelines and was described previously in detail^9,10^.

### Data collection

Outcome was assessed 3 months after discharge using Glasgow Outcome Scale (GOS): GOS 1 = death, GOS 2 = persistent vegetative state, GOS 3 = severe disability, GOS 4 = moderate disability and GOS 5 = good recovery^11^. The participants were usually seen in the neurosurgical outpatient department by a neurosurgical consultant unaware of measurement results, otherwise they were contacted by telephone.

To describe medical history at admission the following factors were considered: arterial hypertension, history of coronary artery disease, arrhythmia, history of cerebrovascular disease, tobacco use, history of asthma/chronic obstructive pulmonary disease (COPD), diabetes mellitus and chronic kidney disease. Further, the severity of SAH was assessed using Hunt–Hess grade, World Federation of Neurologic Surgeons (WFNS) grade and modified Fisher scale, taking into consideration the amount of blood in the initial CT scan^12–14^.

Complications were assessed as follows: cardiac complications (myocardial infarction, hemodynamic relevant arrhythmia, heart failure), pulmonary complications (pulmonary edema, pneumonia, pulmonary embolism), cerebrovascular complications (vasospasm, delayed cerebral ischemia (DCI), need for intra-arterial vasodilator therapy). Cardiac and pulmonary complications were assessed based on the current clinical practice involving symptoms, laboratory and radiological parameters, as well as current guidelines^15^. Vasospasm was diagnosed by daily transcranial Doppler measurements and defined as peak-value increase by > 50 cm/sec/24 h compared to the previous result or a mean value > 120 cm/sec in one of the main supply branches^16^. DCI was defined as a new focal neurological deficit or a cerebral infarction in the presence of vasospasm revealed by radiologic imaging, or both. Other causes of neurological aggravation had to be excluded^17,18^.

### Troponin T measurement

Troponin T was measured daily starting from admission for consecutive 14 days or, if shorter, until discharge from ICU. For troponin T measurements the Roche high-sensitive troponin T Elecsys-assay was used in our laboratory.

### Statistical analysis

Variables are presented as median and interquartile ranges or as mean and standard deviation, as appropriate. Continuous, normally distributed variables were compared using a two-sided Student’s t test, and a Mann Whitney-U-test was used for variables with skewed distributions. Spearman rank test was used to test correlations in non-normally distributed variables. Troponin T values at admission were divided into tertiles. Logistic regression models were applied to assess the relation between initial troponin T values and admission as well as outcome parameters. In order to test whether troponin T is an independent risk factor for worse outcome, a multivariate regression was performed considering age, history of arterial hypertension and WFNS score according to SAHIT core criteria^19^. All statistical tests were done using SPSS statistics 27 (IBM SPSS, Inc.). A p value < 0.05 was considered statistically significant.

## Results

Patients were enrolled between March 2013 and December 2015^9^. Both, outcome and troponin t data, were available in 106 patients. From the initially included 108 patients two were excluded: one due to missing outcome data, the other due to missing troponin T data.

The median age was 55 years and 81% of the patients were female. Almost half of the patients had a history of arterial hypertension and 8% had a history of coronary artery disease. An overview of baseline characteristics is presented in Table 1. 54% of the aneurysms were secured by coiling and 46% were clipped. The median interval from bleeding to surgery was 10 h [6–20 h] (median [interquartile range]). At admission, mean arterial pressure (MAP) was 95 mmHg [83–110 mmHg] and systolic blood pressure was 140 mmHg [120–160 mmHg].

**Table 1:** Patient characteristics at admission.

Characteristics	n (%)
Age—year [median (interquartile range)]	55 (47–69)
Female sex	81 (76)
**Hunt–Hess grade**	
1	14 (13)
2	34 (32)
3	27 (26)
4	23 (22)
5	8 (8)
**WFNS grade**	
1	45 (43)
2	13 (12)
3	8 (8)
4	7 (7)
5	32 (30)
**Modified Fisher scale**	
1	23 (22)
2	10 (9)
3	29 (27)
4	44 (42)
**Aneurysm location**	
AcoA/ACA	38 (36)
MCA	23 (22)
ACoP	14 (13)
ICA	5 (5)
Other	26 (24)
**Medical history at admission**	
Arterial hypertension	50 (47)
Coronary artery disease	8 (8)
Cardiac arrhythmia	8 (8)
Cerebrovascular disease	1 (1)
Asthma/COPD	11 (10)
Tobacco use	32 (30)
Diabetes mellitus	5 (5)
Chronic kidney disease	4 (4)

*WFNS* World Federation of Neurosurgeons, *ACoA* Arteria communicans anterior, *ACA* Arteria cerebri anterior, *MCA* Arteria cerebri media, *ACoP* Arteria communicans posterior, *ICA* Arteria carotis interna, *COPD* Chronic obstructive pulmonary disease.

### Troponin T profiles

**Figure 1:**
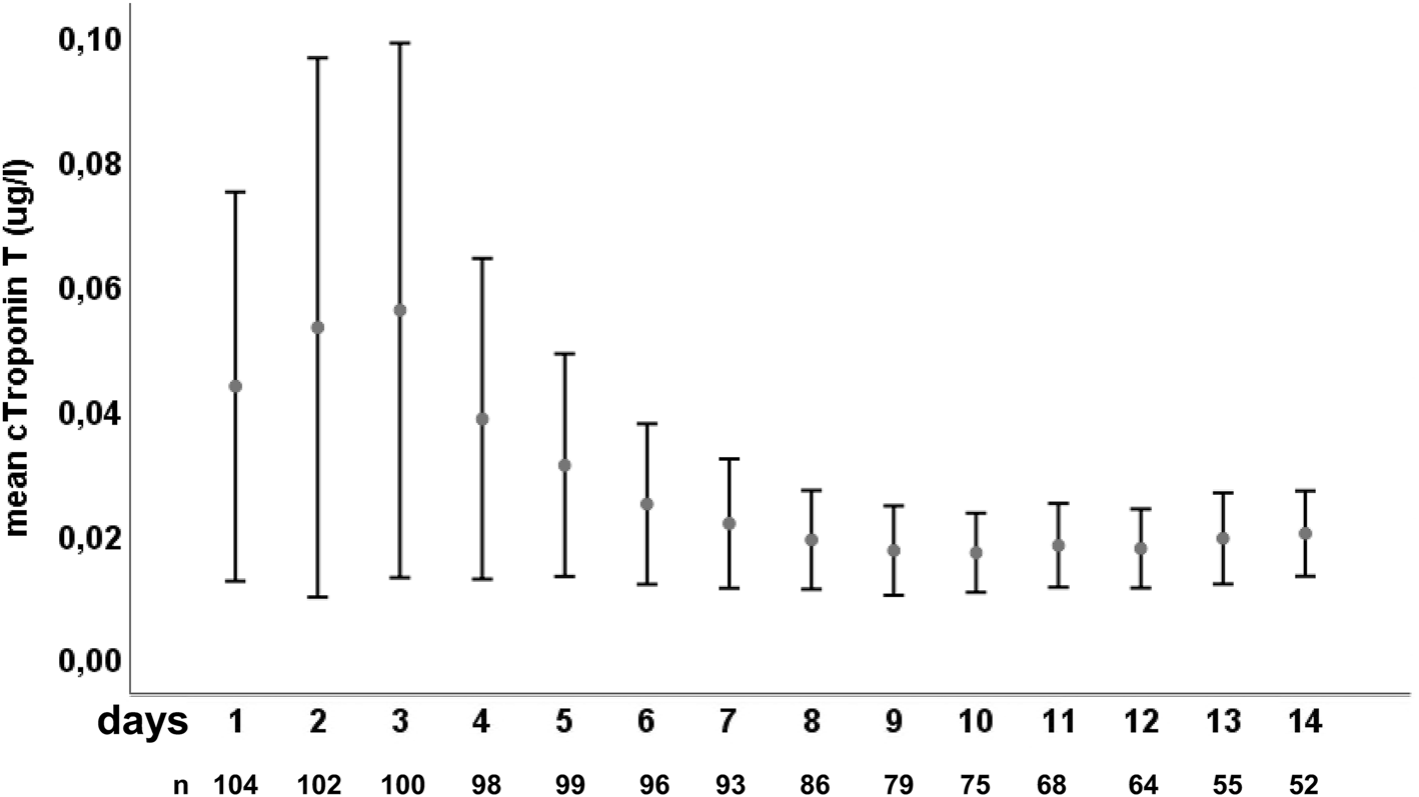
Troponin T levels over time. Mean high sensitive Troponin T levels on the first 14 days after subarachnoid hemorrhage (SAH). Bars represent 95% confidence interval (CI). Day 1 refers to admission.

Troponin T measurements were available in 104 patients (98%) at admission (ICU day 1). The median interval between admission and troponin T peak was 2 days [1–5 days]. In 36% of the patients, the first measurement with a value of 0.011 µg/L [0.005–0.031 µg/L] was the highest. There was a close correlation between the initial troponin T measurement and the peak troponin T value (r = 0.79, p < 0.01). Mean troponin T values were higher (p < 0.001) on days 1–3 (0.054 ± 0.14 µg/L; mean ± SD) compared to days 4–7 (0.027 ± 0.053 µg/L) and days 8–14 (0.016 ± 0.021 µg/L). An overview of troponin T distribution over time is shown in Fig. 1. Troponin T levels at admission were significantly higher in patients with Hunt–Hess grades 3–5 (p < 0.001) and WFNS grades 4–5 (p < 0.001), whereas there was no difference in modified Fisher scale (p = 0.054).

### Outcome

**Figure 2:**
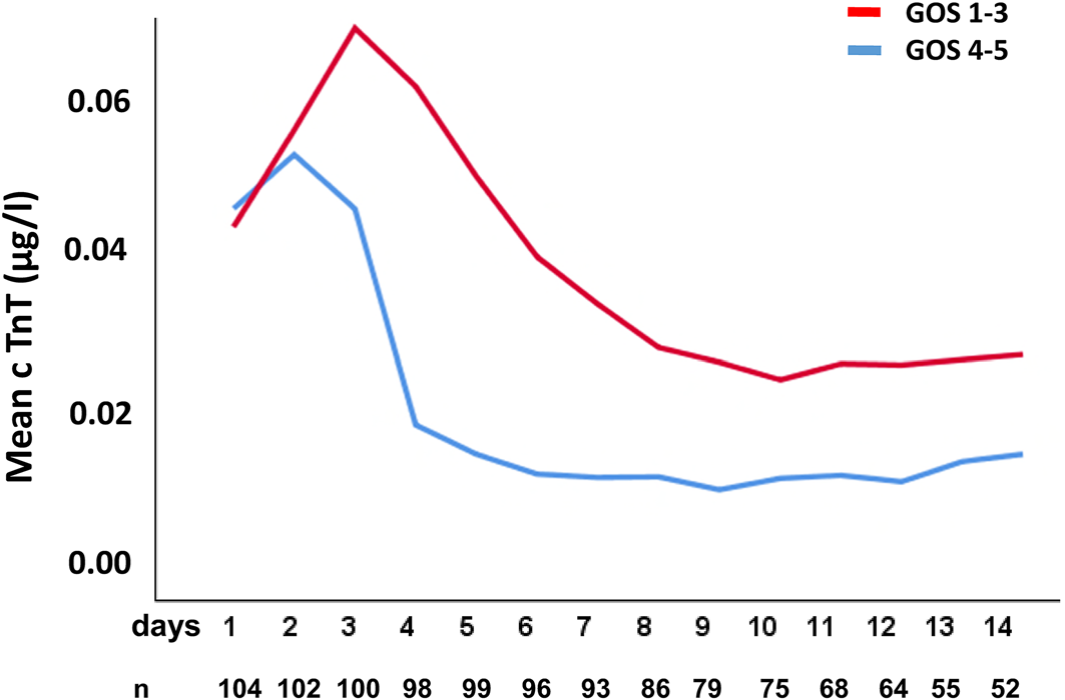
Comparison of Troponin T levels in patients with good and poor recovery. Troponin T levels were significantly higher from admission for further 14 days in patients with moderate to poor recovery (GOS1–3) than in patients with good recovery (GOS4–5), p < 0.05.

GOS was assessed 3 months after discharge: GOS5: 58 patients (55%), GOS4: 10 (9%), GOS3: 12 (11%), GOS2: 12 (11%), GOS1: 14 (13%). There was a significant association between GOS and troponin T values at all 14 days (p < 0.05). Patients with good recovery (GOS4–5) 3 months after admission had lower initial troponin T levels than patients with GOS 1–3 (0.009 µg/L [0.005–0.019] vs. 0.015 µg/L [0.009–0.065], p = 0.013) (Fig. 2). Further, patients with GOS 1 (death) at 3 months had higher troponin T levels at admission than patients who survived after 3 months (GOS 2–5) (0.023 µg/L [0.011–0.045] vs. 0.010 µg/L [0.005–0.022], p = 0.024).

Cardiac complications occurred in 9% of the cases. Ten patients (9%) had a therapy-relevant arrhythmia and one case of congestive heart failure was registered (1%). None of the patients developed a myocardial infarction. Troponin T values at admission were associated with cardiac complications: patients with cardiac complications had higher troponin T levels than those without (0.027 µg/L [0.009–0.071] vs. 0.011 µg/L [0.005–0.025], p = 0.040). 23% of the patients developed pulmonary complications and showed higher troponin T levels at admission compared to those who did not (0.026 µg/L [0.008–0.077] vs. 0.010 µg/L [0.005–0.020], p = 0.007). Vasospasm was observed in 55% of the patients and occurred on day 4 [3–6]. There was no significant difference in initial troponin T levels between those who developed vasospasm and those who did not (0.010 µg/L [0.005–0.029] vs. 0.014 µg/L [0.006–0.032], p = 0.325). Intra-arterial vasodilator therapy was performed in 10% of the cases with no significant differences in initial troponin T levels between patients requiring the therapy and those who did not (0.005 µg/L [0.004–0.065] vs. 0.012 µg/L [0.005–0.03], p = 0.695). Further, 23% of the patients developed DCI on day 5 [4–8]. Similarly, no significant difference was observed in patients developing DCI (23%) and those who did not (initial troponin T: 0.010 µg/L [0.005–0.029] vs. 0.015 µg/L [0.005–0.025], p = 0.830).

In order to assess the relationship between troponin T levels and admission as well as outcome parameters, troponin T values at admission were stratified by tertiles (lowest: troponin T < 0,007 µg/L; intermediate: 0.007–0.019 µg/L; highest > 0.019 µg/L). An overview is shown in Table 2. Troponin T tertiles at admission were significantly associated with death (GOS = 1) or worse outcome at 3 months (GOS = 1–3) (Table 3). After adjustment for age and Hunt–Hess grade, this association was no longer present for death (OR [95% CI] 1.808 [0.717–4.563], p = 0.21) or for worse outcomes at 3 months (GOS1–4) (OR [95% CI] 0.977 [0.507–1.884], p = 0.946). Similarly, there was no association in the multivariate analysis when troponin T was considered as a continuous variable for death (mortality: OR [95% CI] 2.769 [0.102–75.29], p = 0.546, or for unfavorable outcomes at 3 months (GOS1–3) (OR [95% CI] 0.093 [0.003–3.163], p = 0.187)).

**Table 2:** Complications after SAH stratified by Troponin T tertiles at admission.

	Troponin T tertiles at admission			
	Total n(%)	Lowest 0–0.007[µg/L] n = 34	Intermediate 0.007–0.019[µg/L] n = 34	Highest > 0.019[µg/L] n = 36
GOS 1–3 (unfavourable outcome)	38 (37)	7 (21)	13 (38)	18 (50)
GOS 1 (death)	14 (13)	1 (3)	5 (15)	8 (22)
Vasospasm	56 (54)	21 (62)	16 (47)	19 (53)
Intra-arterial vasodilator therapy	11 (10)	6 (18)	1 (3)	4 (11)
Delayed cerebral ischemia (DCI)	22 (21)	9 (26)	5 (15)	8 (22)
Cardiac complications	10 (10)	1 (3)	2 (6)	7 (19)
Pulmonary complications	24 (23)	5 (15)	5 (15)	14 (39)

*GOS* Glasgow Outcome Scale.

**Table 3:** Admission characteristics and outcomes associated with Troponin T.

	n (%)	OR (95% CI) for Troponin T tertiles at admission	P
**Admission characteristics**			
Hunt–Hess grade 3–5	58 (56)	2.703 (1.589–4.598)	< 0.001
WFNS grade 4–5	39 (38)	2.545 (1.480–4.375)	0.001
Modified Fisher Scale 3–4	73 (70)	1.582 (0.934–2.678)	0.088
**Outcome parameters**			
GOS 1–3 (unfavourable outcome)	38 (37)	1.933 (1.153–3.240)	0.012
GOS 1 (death)	14 (13)	2.514 (1.121–5.640)	0.025
Vasospasm	56 (54)	0.837 (0.522–1.342)	0.461
Intra-arterial vasodilator therapy	11 (10)	0.713 (0.328–1.551)	0.394
Delayed cerebral ischemia (DCI)	22 (21)	0.885 (0.498–1.572)	0.677
Cardiac complications	10 (10)	3.094 (1.115–8.591)	0.030
Pulmonary complications	24 (23)	2.076 (1.13–3.814)	0.019

*GOS* Glasgow Outcome Scale.

## Discussion

In this prospective study, we were able to show that troponin T levels measured for the first 14 days are associated with 3 months outcomes assessed by GOS. However, when adjusted for age and Hunt–Hess scores, troponin T levels at admission were not independently predictive of death (GOS 1) or worse outcomes (GOS 1–3) at 3 months.

Cardiac impairment and its impact on outcomes after SAH has been an object of great interest in recent years^20–23^. While there is widely agreement about the mechanism behind these findings involving abnormal sympathetic innervation with an excessive norepinephrine release, their impact on short and long term outcome remains controversial^24^. There are only very few studies who have prospectively evaluated the association between cardiac troponins and outcome after SAH, and only two of them used a high sensitive troponin essay similar to the one in the present study^8,25^. To the best of our knowledge, the present study is the first one prospectively conducting serial hs troponin T measurements for 14 days after admission.

We were able to show that initial troponin T levels are strongly correlated to peak troponin T levels. Additionally, there was an association in the univariate analysis between troponin T levels at admission and severity scores like Hunt-Hess and WFNS grade. Both findings confirm previously published results and support the hypothesis that troponin T elevation is likely to reflect an initial SAH severity with its influence on myocardial dysfunction^26^. Neither vasospasm nor DCI nor the need for intra-arterial vasodilator therapy showed an association with initial troponin T levels in our study. Neither did the modified Fisher scale which is closely related to vasospasm^14^. Due to the disparity of definitions of vasospasm and DCI in previous trials, the results in this field are poorly comparable. While no association between cardiac troponins and vasospasm was found by some investigators^27^, other reported a relation between cardiac troponins and DCI^7,8^. For further research, standardized definitions and approaches are needed in this field and, according to our data, considerably larger sample sizes are warranted in order to draw robust conclusions considering vasospasm and DCI. Further, our work confirms an association between troponin T levels and cardiac as well as pulmonary complications^28^. Finally, while there was an association in the univariate analysis between troponin T levels and 3 months outcomes at all 14 days of measurement, the relation between troponin T tertiles at admission and death as well as worse outcome was no longer present in the multivariate analysis. This finding is in line with previously published retrospective data and even expands those due to the prospective approach^3,5^. Moreover, in a multicenter cohort study, regional wall motion abnormalities (RWMA) after SAH were found to be an independent predictor of outcome after SAH while troponin T elevation was not^7^. Our results confirm the latter observation, but there are also trials suggesting a causal relationship between the early biomarker release such as troponin T and outcome after SAH^8,25^. Further research in a preferably multicenter setting is warranted to clarify the impact of the elevated biomarker levels on the outcome after SAH.

Our study has several limitations. First, this study does not investigate the mechanisms behind the biomarker elevation nor does it consider other cardiac parameters. Second, it is a single center trial with a limited number of patients. Nevertheless, the data were obtained prospectively and continuously. Besides, troponin T as well as 3 months outcome data were collected in all included patients. Third, our follow-up period ended at 3 months. We cannot preclude that a longer follow-up might have led to different results.

Nevertheless, our results add to previous literature as the high sensitive troponin T essay was used as suggested by the current guidelines^29^. We were able to show that troponin T measurements are associated with outcome after SAH as well as cardiovascular and pulmonary complications and might be useful in risk stratification of SAH patients. However, our results suggest that troponin T has limited ability to independently predict outcome after SAH. Further studies are needed to test the utility of hsTroponin T measurements in the early phase after SAH.

## Abbreviations

CT: Computer tomography
DCI: Delayed cerebral ischemia
GCS: Glasgow Coma Scale
GDHT: Goal directed hemodynamic therapy
GOS: Glasgow Outcome Scale
HR: Hazard ratio
MAP: Mean arterial pressure
NSM: Neurogenic stunned myocardium
OR: Odds ratio
RW: Regional wall motion abnormality
SAH: Subarachnoid hemorrhage
SD: Standard deviation
TCD: Transcranial Doppler
WFNS: World Federation of Neurosurgical Societies
